# Roles of second messengers in the regulation of cyanobacterial physiology: the carbon-concentrating mechanism and beyond

**DOI:** 10.1093/femsml/uqad008

**Published:** 2023-02-23

**Authors:** Oliver Mantovani, Michael Haffner, Khaled A Selim, Martin Hagemann, Karl Forchhammer

**Affiliations:** Institute of Biosciences, Department of Plant Physiology, University of Rostock, D-18059 Rostock, Germany; Interfaculty Institute of Microbiology and Infection Medicine, Organismic Interactions Department, Cluster of Excellence ‘Controlling Microbes to Fight Infections’, Tübingen University, D-72076 Tübingen, Germany; Interfaculty Institute of Microbiology and Infection Medicine, Organismic Interactions Department, Cluster of Excellence ‘Controlling Microbes to Fight Infections’, Tübingen University, D-72076 Tübingen, Germany; Department of Protein Evolution, Max Planck Institute for Biology, D-72076 Tübingen, Germany; Institute of Biosciences, Department of Plant Physiology, University of Rostock, D-18059 Rostock, Germany; Interfaculty Institute of Microbiology and Infection Medicine, Organismic Interactions Department, Cluster of Excellence ‘Controlling Microbes to Fight Infections’, Tübingen University, D-72076 Tübingen, Germany

**Keywords:** Synechocystis, cAMP, c-di-AMP, CCM, glycogen, SbtB

## Abstract

Second messengers are a fundamental category of small molecules and ions that are involved in the regulation of many processes in all domains of life. Here we focus on cyanobacteria, prokaryotes playing important roles as primary producers in the geochemical cycles due to their capability of oxygenic photosynthesis and carbon and nitrogen fixation. Of particular interest is the inorganic carbon-concentrating mechanism (CCM), which allows cyanobacteria to concentrate CO_2_ near RubisCO. This mechanism needs to acclimate toward fluctuating conditions, such as inorganic carbon availability, intracellular energy levels, diurnal light cycle, light intensity, nitrogen availability, and redox state of the cell. During acclimation to such changing conditions, second messengers play a crucial role, particularly important is their interaction with the carbon control protein SbtB, a member of the PII regulator protein superfamily. SbtB is capable of binding several second messengers, uniquely adenyl nucleotides, to interact with different partners in a variety of responses. The main identified interaction partner is the bicarbonate transporter SbtA, which is regulated via SbtB depending on the energy state of the cell, the light conditions, and different CO_2_ availability, including cAMP signaling. The interaction with the glycogen branching enzyme, GlgB, showed a role for SbtB in the c-di-AMP-dependent regulation of glycogen synthesis during the diurnal life cycle of cyanobacteria. SbtB has also been shown to impact gene expression and metabolism during acclimation to changing CO_2_ conditions. This review summarizes the current knowledge about the complex second messenger regulatory network in cyanobacteria, with emphasis on carbon metabolism.

## Teaser

In this review we summarized the current knowledge on second messenger signaling in cyanobacteria with special emphasis on their role in the regulation of primary carbon metabolism.

## Second messengers in bacteria

First messengers define extra-cellular signals, whether chemical, biological, or physical that can be detected by membrane or soluble receptors (Kodis et al. 2012). The second messengers represent a class of small molecules and ions that transduce and amplify such extra- and intra-cellular signals to the effector protein(s) to produce a specific response. This class of molecules include a variety of compounds: soluble molecules, composed mainly of nucleotides capable of rapid diffusion inside the cell, lipid messengers for cell wall signaling, ions that allow for signaling between cellular compartments, and free radicals that can transmit signals between adjacent cells (Newton et al. 2016). Second messengers are always present in low concentration that can fluctuate rapidly due to a precise homeostasis of these molecules, which includes a concerted action of enzymes producing and degrading them. The mode of action of second messengers is also very varied, but they always rapidly diffuse inside the cells to quickly reach their target proteins, altering their functions to relay signals (Newton et al. 2016).

Particularly bacteria and eukaryotes make use of second messengers for signaling. However, due to the many differences between the two domains of life, the nature and type of action of second messengers vary. The main reasons are the absence of cellular compartments in bacteria and their broader metabolic diversity. For example, lipid messengers are not as common in bacteria as in eukaryotes, and they mainly function as extra-cellular signaling systems, e.g. for quorum sensing or communication with eukaryotes (Soto et al. 2019). Bacteria employ the use of ions as second messengers as well, but contrary to eukaryotes, they are not used for communication between cellular compartments (Newton et al. 2016). Due to the absence of cellular compartments, nucleotide-type second messengers are more commonly used for communication inside bacterial cells. In fact, many types and functions of nucleotide messengers have already been identified in bacteria, while the same is not always the case in eukaryotes, in which some of the more unusual members of this class have either not been detected at all, or few to no functions have been identified (Schaap, 2013). Furthermore, the nucleotide-type second messengers are often directly impacting gene expression regulation in bacteria, because some of them can bind to riboswitches thereby tuning the stability or translation of specific mRNAs (Wachter, 2010).

## Second messengers in cyanobacteria

In contrast to heterotrophic model bacteria such as *Escherichia coli* and *Bacillus subtilis*, photoautotrophic cyanobacteria are constantly exposed to alternating day-night light regimes, which requires a permanent metabolic switch between autotrophic CO_2_ fixation via the Calvin–Benson cycle during the day and heterotrophic-like carbon catabolism during the night. During daytime, newly fixed CO_2_ is used for anabolic reactions, producing the building blocks for cell growth and, in addition, for building up organic carbon reserves such as glycogen. During nighttime, glycogen is metabolized using mainly the oxidative pentose-phosphate pathway to provide reduction equivalents for energy conserving respiration (Makowka et al. 2020). The constant diurnal switch between day and night metabolism promoted the evolution of a circadian clock in cyanobacteria, which helps to adjust the metabolism toward the upcoming light or dark conditions. Thus, the sophisticated network of regulatory processes for cyanobacterial metabolism involves sensing of the redox, energy, carbon, and nitrogen status as well as the circadian clock (Forchhammer, 2004; Welkie et al. 2019; Gurrieri et al. 2021). In addition, it has been shown that diverse second messengers change their concentrations in response to environmental fluctuations in cyanobacterial cells (reviewed in Agostoni and Montgomery, 2014), which will be exemplified in the upcoming paragraphs.

### AMP, ADP, and ATP

ATP is the most common energy-carrying molecule in all organisms, used for the majority of energy-requiring cellular processes. Energy is released by the hydrolysis of the γ- or β-phosphate groups, yielding ADP or AMP, respectively, which are then used to regenerate ATP anew. The synthesis of ATP is mainly performed by F-type ATP synthase, powered by membrane potential generated via photosynthetic electron flow during the day or via respiration during the night (Song et al. 2022). Adenylate kinase, which performs the reversible reaction 2 ADP ↔ 1 ATP + 1 AMP, allows energy buffering through interconversion of the adenyl-nucleotide pools (Nitschmann and Peschek, 1986). Because of this, while ATP, ADP, and AMP are not categorized as second messengers, the ratios of ATP/ADP and more importantly of ATP/AMP constitute a signal for the energy state of the cells, capable of influencing many different regulatory proteins to cause an effect similar to second messengers. In the photoautotrophic cyanobacterial cell, in which the cellular energy levels are mainly dependent on light availability, the ratios of ATP, ADP, and AMP strongly influence anabolic metabolism, especially those of carbon and nitrogen assimilation (Mantovani et al. 2022; Selim et al. 2019). The canonical PII signaling protein (GlnB) serves as a central signal processor that combines the sensing of the ATP/ADP ratio with the sensing of the central carbon/nitrogen status metabolite 2-oxoglutarate (Fokina et al. 2010), thereby regulating central reactions in nitrogen and carbon assimilation (Forchhammer and Lüddecke, 2016; Forchhammer and Selim, 2020; Forchhammer et al. 2022).

### Cyclic AMP (cAMP)

Adenosine 3′5′-cyclic AMP (cAMP) represents the first discovered and best investigated second messenger for intra-cellular signaling in all forms of life. The cAMP molecule is generated from a molecule of ATP by the enzyme adenylate cyclase and degraded through hydrolysis by phosphodiesterases (PDEs). Due to the importance of cAMP, multiple adenylate cyclases exist within each organism, divided in different classes depending on their amino acids sequence, found either in the cytosol or associated to the membrane (McDonough and Rodriguez, 2011). Many cellular responses are regulated by cAMP through different mechanisms. Among bacteria, most cAMP-dependent effects are transduced by the cAMP receptor protein (CRP), a transcription regulator mediating catabolite repression, capable of affecting many genes related to carbohydrate utilization (Görke and Stülke, 2008). However, while the cAMP-CRP complex has been seen to be a key regulator in carbon catabolism among heterotrophic bacteria, the same is not the case in cyanobacteria, in which not many functions for CRP have been identified yet (Xu and Su, 2009).

**Figure 1.**
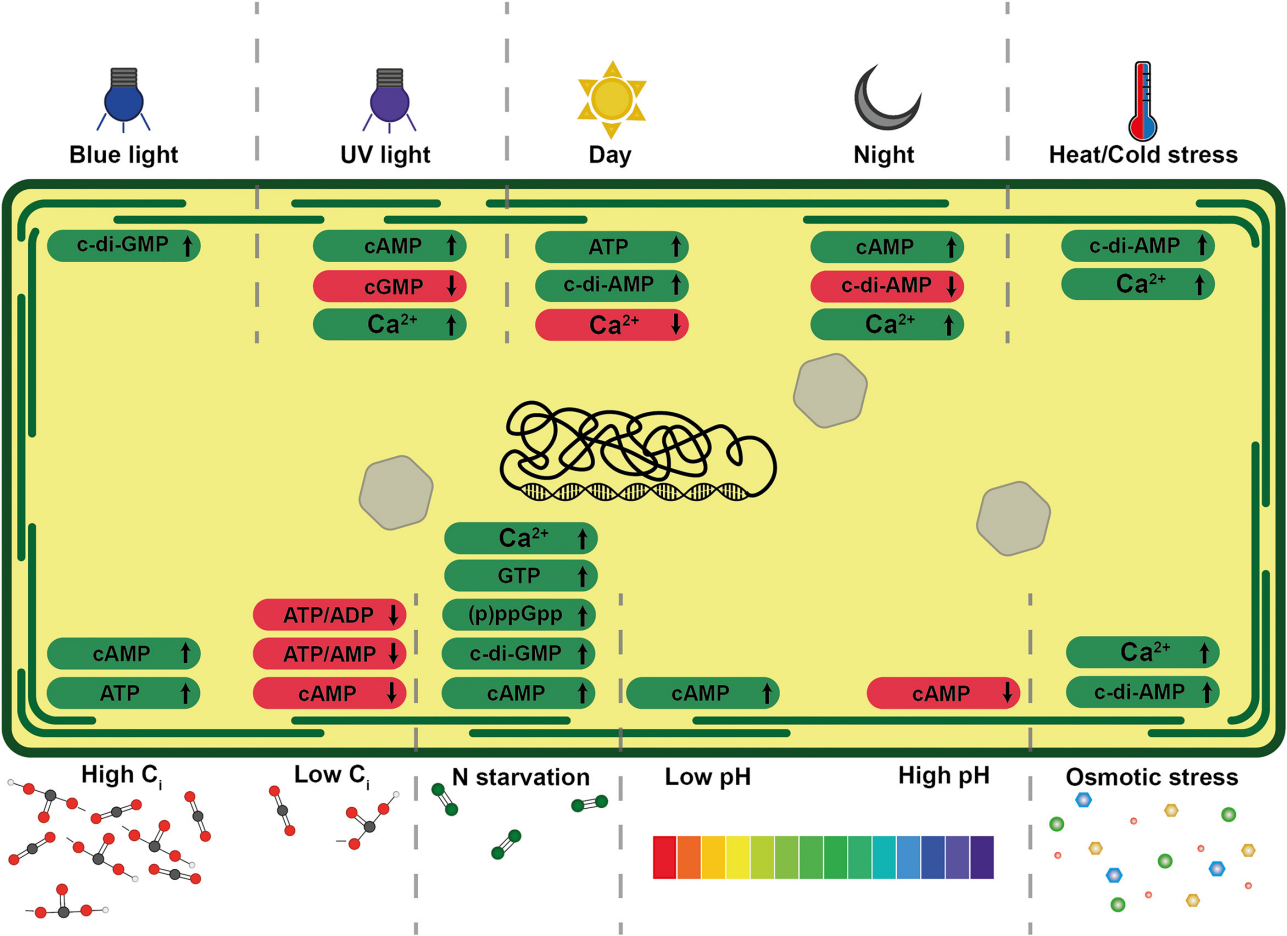
Schematic representation of reported environmental stimuli on the concentrations of the most important second messengers in cyanobacteria (as shown in text). Above and below the cyanobacterial cell, the specific stimuli, first messengers are represented. The effect of each is shown inside the cell, with green and red highlights indicating an increase and decrease in the concentration of the specific second messengers, respectively. In the top, from the left, the effect of blue light, UV light, day/night cycle, and temperature stress are shown. In the bottom, from the left, the signaling of high/low inorganic carbon, nitrogen starvation, low/high pH, and osmotic stress are displayed. In the center of the cell, DNA and carboxysomes are depicted as targets of second messenger signaling.

Among cyanobacteria, it has been reported that cAMP levels are affected by a variety of environmental factors, such as light, pH, oxygen, nitrogen, and inorganic carbon levels (e.g. Yoshimura et al. 2000) (Fig. 1). The large number of factors that affect the levels of this second messenger reflect the variety of identified functions influenced by cAMP levels, such as the regulation of motility in response to light at protein and transcriptional level (Ohmori and Okamoto, 2004), as signal for nutrient deficiency (Francko and Wetzel, 1981), and the regulation of rehydration after desiccation (Imashimizu et al. 2005). More recently, we showed that cAMP also plays a role in the sensing of carbon status in cyanobacteria due to its binding with the carbon regulator protein SbtB (Selim et al. 2018; more details are given below). The regulation of carbon metabolism by cAMP also includes the cyanobacterial CRP, SyCRP, particularly in the regulation of the CCM (Bantu et al. 2022).

### cGMP

While in eukaryotes cGMP plays an important role as second messenger in many processes, in bacteria not many targets for cGMP have been identified, and it appears that the di-cyclic variant c-di-GMP is of more significance (Pesavento and Hengge, 2009). This situation is similar among cyanobacteria. It has been shown that cGMP is declining under UV stress conditions in the often used model strain *Synechocystis* sp. PCC 6803 (hereafter *Synechocystis*), while at the same time the amount of cAMP is rather increasing. A PDE encoded by *slr2100* was shown to be specific for cGMP degradation, and its mutation resulted in a higher UV sensitivity due to impaired repair of photosystem II (Cadoret et al. 2005). Furthermore, the protein Cya2 (encoded by *sll0646*) was verified as a cGMP synthesis enzyme in *Synechocystis* (Ochoa De Alda et al. 2000). It is also interesting to note that several light-sensing proteins in cyanobacteria bear cGMP-specific PDE domains (e.g. Fushimi and Narikawa, 2019), which makes it likely that cGMP as well as c-di-GMP (as shown below) is involved in light sensing and cyanobacterial motility (Fig. 1).

### c-di-AMP

The di-cyclic AMP (c-di-AMP) is a nucleotide-type second messenger that was recently discovered. Until now it has only been found in prokaryotes, where it seems to be particularly important for potassium homeostasis and osmoregulation (Stülke and Krüger, 2020). The second messenger c-di-AMP is produced from two molecules of ATP through the di-adenylate cyclase (Dac) and is degraded by specific PDEs. Contrary to cAMP, usually one Dac enzyme exists in each bacterium. In the model cyanobacterium *Synechocystis* the only Dac is encoded by the gene *sll0505* (Selim et al. 2021b), while the *slr0104* gene is encoding the PDE (Agostoni et al. 2018). Not many effectors for this second messengers have been identified yet, but one of the clearly discovered modes of actions for c-di-AMP is through riboswitches (Nelson et al. 2013), which have been verified in cyanobacteria as well (Mantovani et al. 2022). In *Synechocystis*, this c-di-AMP-dependent riboswitch regulates the *slr0753* gene that codes for a putative chloride efflux transporter (Kobayashi et al. 2006). These findings are consistent with a role of c-di-AMP in osmotic tolerance in *Synechocystis* (Agostoni et al. 2018; Selim et al. 2021b).

The functions of c-di-AMP in cyanobacteria are being investigated thoroughly in recent years, and among the identified roles are acclimation to diurnal day/night cycle and glycogen metabolism (Rubin et al. 2018; Selim et al. 2021b), salt stress acclimation (Agostoni et al. 2018; Zarrella and Bai 2021; Selim et al. 2021b), exopolysaccharide secretion (Peng et al. 2016), and nitrogen metabolism through the involvement in the resuscitation of *Synechocystis* cells from long-term chlorosis after nitrogen starvation condition (Klotz et al. 2016; Selim et al. 2021b) (Fig. 1). The regulation of glycogen metabolism is mediated through the interaction with the SbtB signaling protein and the glycogen branching enzyme GlgB in a c-di-AMP-dependent manner (Selim et al. 2021b; for more details see below).

### c-di-GMP

The second messenger di-cyclic GMP (c-di-GMP) has been shown to be involved in regulation of biofilm formation, virulence, and decision making between sessile or planktonic lifestyle in many bacteria (Jenal et al. 2017). It is synthesized by diguanylate cyclases that contain a GGDEF domain and is cleaved by PDEs. In cyanobacterial genomes several proteins have been identified containing GGDEF domains and are thus potentially capable to synthesize c-di-GMP. These domains are often found in photoreceptors implying that c-di-GMP is of high importance for light sensing and light-dependent motility in cyanobacteria such as *Synechocystis* and others (Wallner et al. 2020; Nakane et al. 2022). For example, it has been verified that the *Synechocystis* protein Slr1143 is an active diguanylate cyclase that interacts with the red-light photoreceptor Cph2 (Angerer et al. 2017). In addition to light-dependent movement, c-di-GMP is also involved in cell aggregation and biofilm formation in different cyanobacteria (Agostoni et al. 2016; Enomoto et al. 2018). Furthermore, this second messenger is important for cell differentiation among cyanobacteria, because it plays a role in the development of N_2_-fixing heterocysts in *Anabaena* sp. PCC 7120 (Huang et al. 2021) (Fig. 1).

### (p)ppGpp

The alarmone (p)ppGpp is a well-known second messenger promoting the stringent response among bacteria, i.e. the massive downregulation of transcription under starvation. This alarmone has been early detected in cyanobacterial cells as well, in which its content is correlating with stable RNA synthesis under different light regimes (e.g. Mann et al. 1975). In addition to light, (p)ppGpp contents also varied under different nitrogen supply in cyanobacteria (Wood and Haselkorn 1980; Friga et al. 1981), and the alarmone seems to be also somehow involved in the differentiation of N_2_-fixing heterocysts in *Anabaena* sp. PCC 7120 (Zhang et al. 2013). Recently, it was clearly shown that (p)ppGpp is crucial for the light/dark acclimation in *Synechococcus elongatus*. The amount of ppGpp increases after transfer into darkness and its absence resulted in the inability of diurnal growth. The alarmone signal inhibits the expression of many genes in darkness and has also impact on the degradation of some macromolecular compounds in *S. elongatus* (Hood et al. 2016; Puszynska and O’Shea 2017) (Fig. 1). There are also hints that ppGpp can participate in the response to oxidative stress caused by environmental factors such as high light (Jin et al. 2020).

### Ca^2+^

Calcium (Ca^2+^) ions are another important second messenger among cyanobacteria (Agostoni and Montgomery 2014). It operates in metabolic signaling and/or cellular differentiation. The intracellular Ca^2+^ concentrations are tightly controlled via Ca^2+^ transporters and via Ca^2+^-binding proteins to keep the free cytoplasmic Ca^2+^ in low nM concentrations. The increase in the intracellular Ca^2+^ concentrations rapidly transmits signals to initiate developmental processes such as heterocyst differentiation. The first Ca^2+^-binding protein CcbP (*alr1010*) was identified within the filamentous, multicellular cyanobacterium *Anabaena* sp. PCC 7120 (Zhao et al. 2005). Physiological and biochemical studies indicated that high intracellular Ca^2+^ concentrations represent a low nitrogen/high carbon signal inducing heterocyst differentiation. Structural analysis of CcbP revealed two distinguishable Ca^2+^-binding sites, one with high affinity in the µM range and another with low affinity in the mM range (Hu et al. 2011). Replacement of aspartate_38_ to alanine (D38A) in the high affinity Ca^2+^-binding site abolished the ability of CcbP to tightly bind Ca^2+^ and thereby to control heterocyst differentiation.

Mining cyanobacterial genomes identified a small protein with two characteristic Ca^2+^-Sensor EF-hand domains (CSE protein), which is exclusively encoded in genomes of multicellular cyanobacteria. The CSE-encoding gene *asr1131* is strongly downregulated during nitrogen limitation, a condition inducing heterocyst formation in *Anabaena* sp. PCC 7120 (Walter et al. 2019). In contrast, low CO_2_ conditions caused rapid upregulation of *asr1131*, to trap the free intracellular Ca^2+^ ions, keeping the cytoplasmic Ca^2+^ at low levels. These observations clearly imply that CSE is under the control of C- and N-availability. Physiological analysis revealed that the Ca^2+^ signaling via the CSE protein is required for the regulation of photosynthesis, the correct assembly of phycobilisomes, and downstream energy and electron transfer routes, thereby affecting the overall cell fitness. Moreover, CSE is needed for proper differentiation and full function of heterocysts (Walter et al. 2020).

In addition to the role of Ca^2+^ in the regulation of the C/N homeostasis among filamentous cyanobacteria, *in vivo* studies revealed that the PII signaling protein and the transcription factor NtcA (nitrogen control protein A) are required to trigger the transient Ca^2+^ signal (Leganés et al. 2009). Analogous to NdhR (as shown below), the transcription regulator NtcA is involved in the activation of many genes involved in nitrogen assimilation in cyanobacteria under N-limiting conditions, which are mainly sensed and transduced by the PII protein to achieve C/N homeostasis (Forchhammer and Selim 2020). Furthermore, Ca^2+^ appears to regulate motility in cyanobacteria, as both hormogonia differentiation and directional motility were found to be under the control of intercellular Ca^2+^ waves. In multicellular *Nostoc* spp., pivotal roles of Ca^2+^ in fast–stimulus–responses have been frequently associated with daily light-to-dark transitions, temperature fluctuations (heat or cold shock), and salt or osmotic stress (Agostoni and Montgomery 2014).

## Carbon-concentrating mechanism in cyanobacteria

### Overview

All oxygenic phototrophic organisms, including cyanobacteria use the Calvin–Benson cycle and its carboxylating enzyme ribulose 1,5-bisphosphate carboxylase/oxygenase (RuBisCO) for the fixation of CO_2_ to form two molecules of 3-phosphoglycerate (3PGA). However, RuBisCO catalyzes a competitive side reaction with O_2_, leading to formation of 2-phosphoglycolate (2PG) that must be salvaged via the photorespiratory pathway, in which two 2PG molecules are recycled to one 3PGA molecule with concomitant release of CO_2_ and ammonia (Hagemann et al. 2016; Busch 2020). During geological times, oxygenic photosynthesis led to a massive decrease in the atmospheric CO_2_ and increase in the atmospheric O_2_ concentrations. In current atmospheric conditions (∼400 ppm CO_2_, 21% O_2_), about every fourth reaction of RuBisCO is with O_2_. Moreover, in aquatic habitats, the solubility of CO_2_ is low and the amount of inorganic carbon (Ci; CO_2_ and its dissolved form bicarbonate—HCO_3_^−^) varies largely with temperature, salinity, and pH. To adapt to these unfavorable conditions, cyanobacteria evolved an efficient inorganic carbon-concentrating mechanism (CCM) that concentrates CO_2_ and suppresses the wasteful side reaction of RuBisCO with O_2_. The timing of the earliest appearance of cyanobacterial CCM is controversial (400–2000 million years ago; Badger and Price 2003; Kupriyanova et al. 2013). The well conserved beta-carboxysomes likely evolved quite early before the large cyanobacterial radiation occurred (Melnicki et al. 2021).

**Figure 2.**
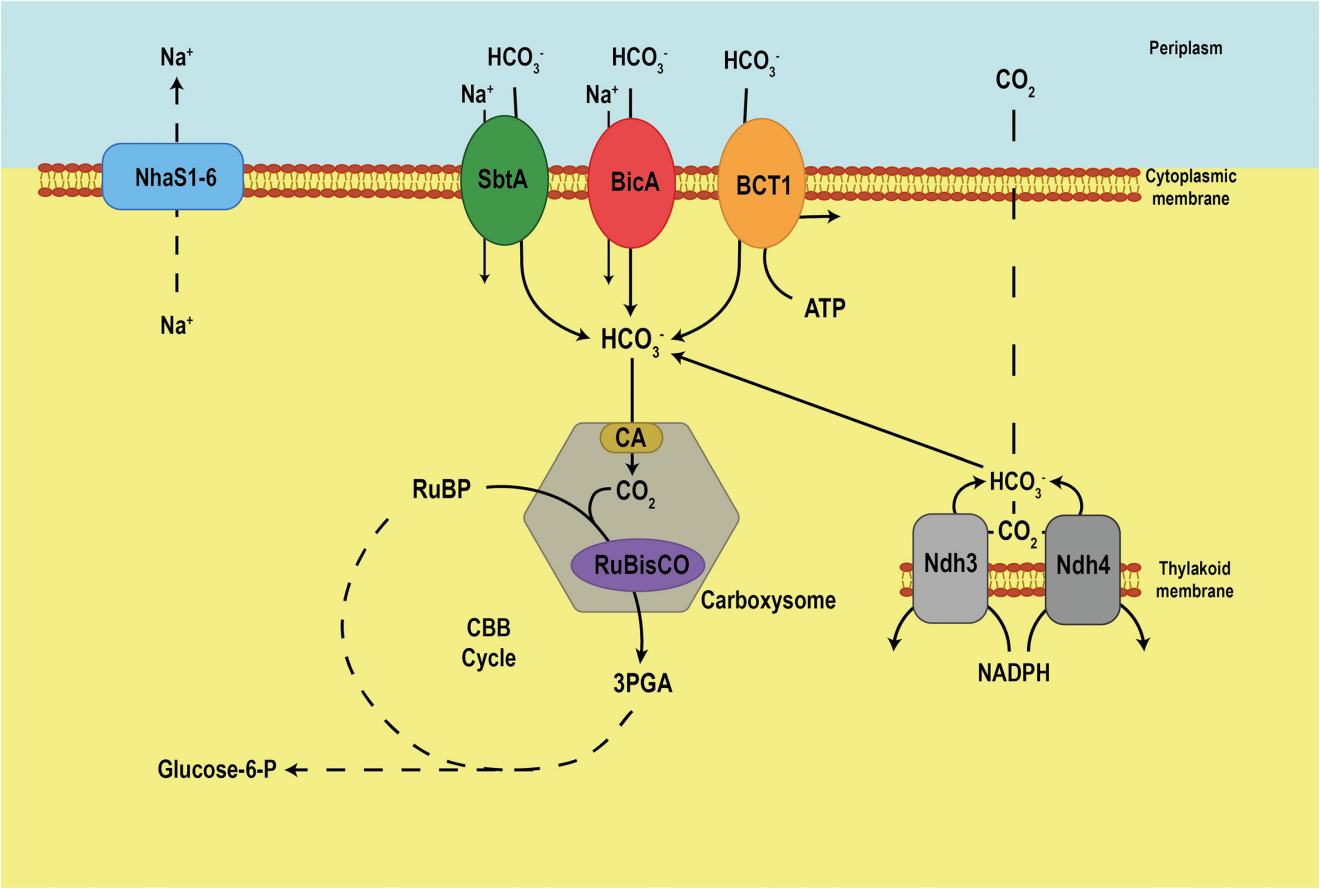
Schematic representation of the cyanobacterial inorganic carbon-concentrating mechanism (CCM). Three bicarbonate transporters (SbtA, BicA, and BCT1) and the Na^+^-gradient restoring different Na^+^/H^+^ antiporters (NhaS1-6) are located at the plasma membrane. Together with two CO_2_-hydrating systems on thylakoid membranes they accumulate high internal bicarbonate levels. Bicarbonate is diffusing into the carboxysome, in which CO_2_ is released by carbonic anhydrase (CA) thereby promoting the carboxylation reaction of RuBisCO.

Cyanobacteria possess a so-called biophysical CCM, which initially accumulates high concentrations of bicarbonate inside the cell. In contrast to CO_2_, this ion can be actively transported and cannot easily escape through membranes from the cell (Fig. 2). Cyanobacteria utilize three bicarbonate transporters, one for the primary active bicarbonate transport via the ABC transporter BCT1 (Omata et al. 1999) and two Na^+^-bicarbonate symporters SbtA (Shibata et al. 2002) or BicA (Price et al. 2004). SbtA and BCT1 are induced under low Ci conditions, whereas BicA is rather constitutively expressed. The BCT1 transport system is controlled by the transcription factor CmpR, which becomes activated via the binding of the metabolites 2PG and ribulose 1,5 bisphosphate that accumulate under low Ci conditions (Nishimura et al. 2008; the regulation of *sbtA* is explained in the next chapter). In addition, two specialized NDH1 complexes can hydrate inward-diffusing CO_2_ or CO_2_ escaping from the carboxysome and thereby contribute to the accumulation of bicarbonate inside the cytoplasm (Shibata et al. 2001; Hagemann and Kaplan 2020). Overall, these five systems actively concentrate bicarbonate within the cells, which then diffuses into the bacterial microcompartment carboxysome, where it is converted to CO_2_ by carbonic anhydrase (CA) to generate a high concentration of CO_2_ around RuBisCO (Kaplan and Reinhold 1999; Rae et al. 2013; Burnap et al. 2015; Hagemann et al. 2021). Carboxysomes are composed of about 10 proteins, including a self-assembling proteinaceous sheath that encapsulates RuBisCO and CA as well as proteins that are required for assembly and organization of RuBisCO (Kerfeld et al. 2018; Lechno-Yossef et al. 2020; Liu 2022). Mutations that abolish bicarbonate accumulation or carboxysome function lead to loss of the ability to grow at ambient CO_2_ concentrations, but can be rescued at enhanced CO_2_ levels (e.g. Marcus et al. 1992; So et al. 2002; Xu et al. 2008).

### Bicarbonate Transporter SbtA and Regulatory Protein SbtB

The SbtA protein represents the main bicarbonate transporter under low Ci conditions. It has been initially identified in the model cyanobacterium *Synechocystis* (Shibata et al. 2002). This transporter is highly conserved among cyanobacteria, however, picoplanktonic, so-called alpha-cyanobacteria contain a paralog without proven bicarbonate transport function. It is worth mentioning that SbtA-like proteins also exist in numerous heterotrophic bacteria (von Rozycki et al. 2004). SbtA functions as a sodium/bicarbonate symporter (Shibata et al. 2002) and its expression is highly stimulated under limiting Ci conditions due the inactivation of the global carbon repressor protein NdhR (e.g. Klähn et al. 2015). Recent structural investigations of SbtA revealed that the protein forms a homotrimer, in which each subunit can symport one bicarbonate together with one sodium ion (Fang et al. 2021; Liu et al. 2021).

In *Synechocystis* as in many other cyanobacteria, the *sbtA* gene forms an operon with *sbtB* that codes for a small conserved protein, initially annotated as protein of unknown function showing some structural similarities to PII signaling proteins. First functional insights into SbtB were obtained from experiments expressing the *sbtAB* operon in an *E. coli* mutant that lacks CA activity and thus depends on bicarbonate uptake, allowing characterization of the SbtA-dependent bicarbonate transport activity. These studies revealed that SbtB had a negative impact on SbtA activity in this heterologous host (Du et al. 2014). As described below in more detail, SbtB interacts with SbtA. This interaction is affected by cAMP (Selim et al. 2018) and modulates the cyanobacterial CCM. The structure of the SbtB-SbtA complex and its potential impact on the transport activity has recently been published (Fang et al. 2021; Liu et al. 2021).

## Role of second messengers in the regulation of the CCM

Due to the many components and important function of the CCM, this mechanism needs to be finely regulated to allow for a quick acclimation to environmental changes. Because of this, second messengers are needed for the regulation of the CCM and the downstream carbon metabolism. While the regulatory network of the CCM is still not entirely known, one of the main candidates among second messengers is cAMP. In fact, the activity of the main soluble adenylate cyclase (sAC) Cya1 (*slr1991*) in *Synechocystis*, responsible for the production of most of the cAMP (Terauchi and Ohmori 1999), was seen to be enhanced in response to increasing CO_2_ (Hammer et al. 2006) and/or bicarbonate concentrations (Steegborn et al. 2005). These findings strongly supported the view that cAMP acts as a signal for high Ci availability, which was proven later (Selim et al. 2018; Bantu et al. 2022). The same was observed for the sAC CyaB1 in *Anabaena* sp. PCC 7120 (Cann et al. 2003; Hammer et al. 2006). Moreover, the synthesis of the second messenger cAMP by sAC seems to be regulated by another second messenger, Ca^2+^ ions that also represent a high Ci signal at least in filamentous strains (as shown in section Ca^2+^). The binding of Ca^2+^ to the cyanobacterial sAC directly mediates the ATP binding and thereby the cAMP synthesis (Steegborn et al. 2005). In addition to cAMP, c-di-AMP has been also identified to play a role in the regulation of the CCM (Selim et al. 2021b; Mantovani et al. 2022).

Despite the broad regulatory role played by second messengers, central metabolites of the primary carbon/nitrogen metabolism are also used as metabolic status reporters to regulate the CCM, such as 2PG as signal for low CO_2_ availability or 2-oxoglutarate as high CO_2_ signal. These metabolic signals are crucial for the activation/inactivation of transcriptional factors mainly NdhR regulating the expression of CCM-related genes such as *sbtAB* (Jiang et al. 2018; Forchhammer and Selim 2020; Hagemann et al. 2021).

### SbtB Is a Major Receptor for Adenyl Nucleotides

**Figure 3.**
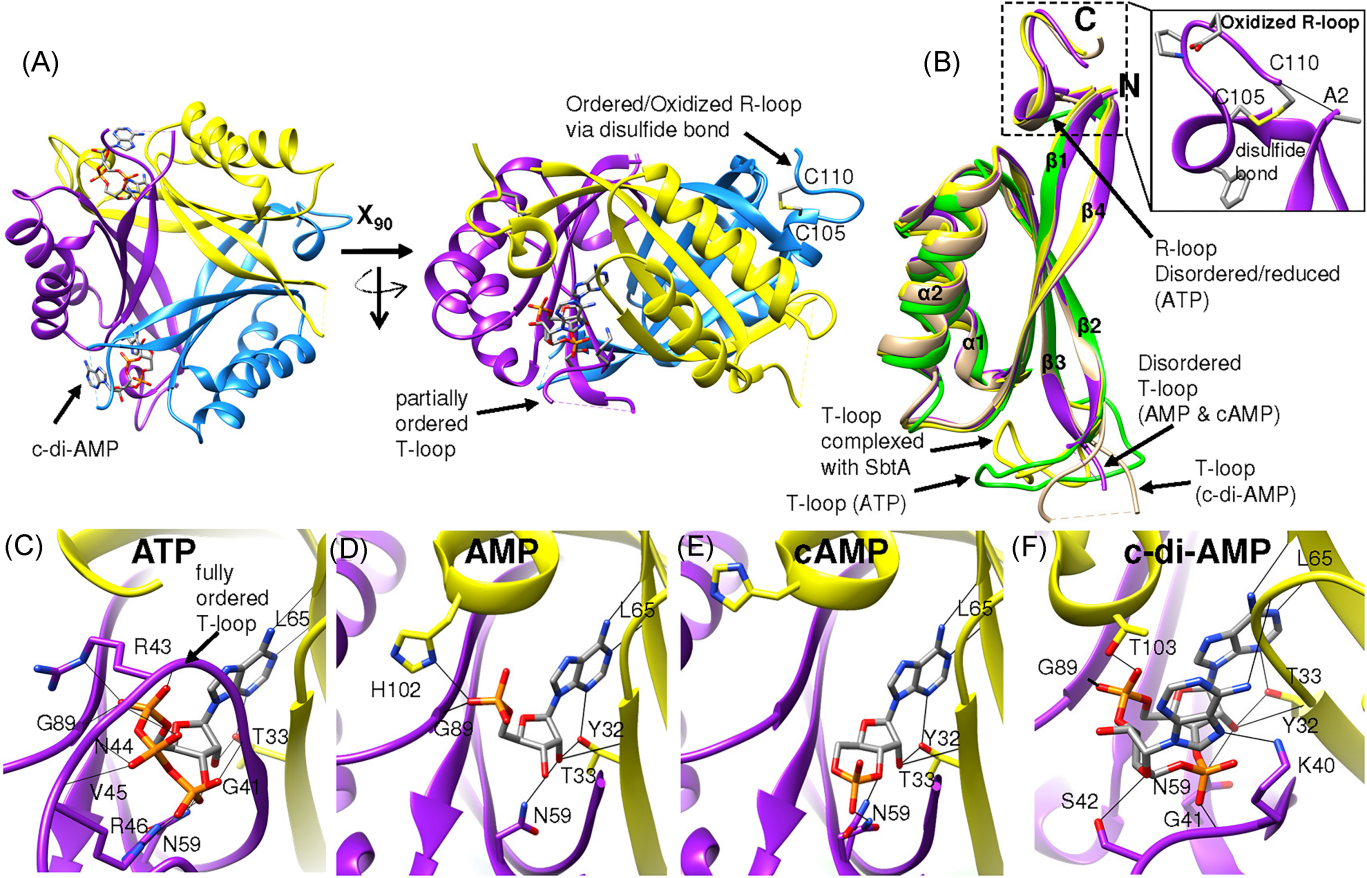
Structural insights and sensing properties of SbtB protein. **(A)** Top and side view on the trimeric architecture of the SbtB-c-di-AMP complex (PDB:7OBJ; in ribbon representation with different color for each monomer). The SbtB structure reveals the typical ferredoxin-like fold of the PII superfamily, with nucleotide-binding pockets located between the clefts of the subunits. The characteristic structural motifs (T- and R-loops) are indicated. The large flexible T-loop is partially ordered and therefore not fully resolved. The oxidized R-loop forms a hairpin via disulfide bond between Cys_105_ and Cys_110_ residues. **(B)** Superposition of monomeric subunits of SbtB complexed with either AMP (violet; PDB:5O3R), c-di-AMP (brown; PDB:7OBJ), ATP (green; PDB:7R31), or SbtA (yellow; PDB:7EGK), showing the high flexibility of the T-loop with different conformations. The T-loop is the major target-binding element of PII superfamily proteins. The secondary structure elements and characteristic structural motifs are indicated. (Inset) The oxidized SbtB R-loop (C_105_GPEGC_110_) motif with the disulfide bond is highlighted. The reduced R-loop in the SbtB-ATP complex (green; PDB:7R31) is disordered, while the T-loop is fully structured. **(C–F)** Close-up of the nucleotide binding site of SbtB-ATP **(C)**, SbtB-AMP **(D)**, SbtB-cAMP **(E)**, and SbtB-c-di-AMP **(F)**. The relevant residues for nucleotide binding and the nucleotides are shown in stick representation, with O, N, and P atoms colored in red, blue, and orange, respectively. H-bonds are indicated by black lines. The R_43_xxR_46_ motif coordinates the ATP phosphate groups. For more details, see Selim et al. (2018; 2021b; 2023a).

Biochemical analysis of SbtB, the protein that is coexpressed with SbtA, provided important clues to elucidating an intricate network of interactions involved in carbon-homeostasis in cyanobacteria. Structural analysis of the SbtB protein from *Synechocystis* and other cyanobacteria confirmed the high similarity to PII signaling proteins. However, in contrast to canonical PII proteins, SbtB not only binds the adenyl nucleotides ATP and ADP, but also AMP as well as the second messengers cAMP and c-di-AMP (Selim et al. 2018; 2021b; Kaczmarski et al. 2019). Meanwhile, the structures of the various SbtB-adenyl-nucleotide complexes were solved (Fig. 3). The binding mode of the adenosine moiety is sterically almost identical to canonical PII proteins, although other amino acid side chains are involved. Like canonical trimeric PII proteins, the SbtB trimer exposes a large disordered loop (T-loop) from each subunit, which protrude from the effector binding site and may adopt folded structures upon binding of certain interaction partners. As in most PII structures, the T-loop was also disordered in SbtB structures in complexes with AMP or cAMP.

Surprisingly, attempt to cocrystallize SbtB with ATP and ADP failed, only SbtB-AMP complexes were observed. Recent work demonstrated that SbtB possesses intrinsic apyrase (diphosphohydrolase) activity, converting the adenosine nucleotides into the stable SbtB-AMP complex (Selim et al. 2023a). The cyclic adenyl nucleotides bind in the same pocket as linear adenyl nucleotide with the adenosine and ribose moieties occupying the same sites (Fig. 3). Unsurprisingly, the phosphate binding mode differed for cAMP and c-di-AMP. The c-di-AMP binding involved additional contacts with the base of the T-loop causing a partial structuring and ordering of the loop (Selim et al. 2021b). The 3D-structure analyses revealed an additional distinctive feature of *Synechocystis* SbtB. Its C-terminus formed a hairpin loop that was stabilized by a disulfide bridge between two cysteine residues located in the C-terminal C_105_GPxGC_110_ motif (Fig. 3B), resembling a redox-sensitive module (as shown below).

### SbtB Is involved in cyanobacterial CCM

Insights into the physiological function of SbtB were obtained by analyzing the phenotype of SbtB deficient mutants in *Synechocystis*. These mutants showed impaired regulation of the CCM in response to changes in ambient Ci supply. Whereas wild-type cells change their Ci-uptake affinity according to the ambient CO_2_ supply, with low-CO_2_-acclimated cells exhibiting high Ci-uptake affinity and high-CO_2_-acclimated cells showing reduced affinity, the SbtB deficient mutant constitutively resided in the high Ci-affinity state of low-CO_2_-acclimated cells. Under ambient air conditions, the SbtB mutant also grew slower than the wild type and was unable to cope with fluctuating Ci and light intensities (Selim et al. 2018). The perturbation in Ci-metabolism caused by *sbtB* mutation suggested a functional link with the bicarbonate transporter SbtA. The reported negative effect of SbtB on SbtA activity in the heterologous *E. coli* expression system (Du et al. 2014) implied a model according to which SbtB acts as negative regulator of SbtA activity, in analogy to the negative regulation of ammonium channel AmtB by the canonical PII protein GlnK (Conroy et al. 2007). In agreement with this assumption, SbtB was found to be membrane associated in an SbtA-dependent manner.

Recent structural work revealed the structure of the sodium-dependent bicarbonate transporter SbtA in complex with SbtB (Fang et al. 2021; Liu et al. 2021). Overall, the structure resembles the GlnK-AmtB paradigm of PII-transporter complexes. Cryo-electron microscopy resolved a trimeric SbtA transporter that faces at its cytoplasmic side the SbtB-AMP trimer complex with its T-loops inserting into the cytoplasmic cavity of SbtA. AMP is required for the T-loop to adopt the SbtA-binding structure (Fang et al. 2021; Liu et al. 2021), consistent with the positive effect of AMP on membrane association of SbtB (Selim et al. 2018). The binding mode of cAMP is incompatible with the folding of the T-loop in the SbtA complex, explaining why cAMP prevents the formation of the SbtA-SbtB complex at high CO_2_ conditions (Fang et al. 2021; Selim et al. 2023a). In two phylogenetically distant cyanobacteria, *Cyanobium* sp. PCC 7001 and *Synechococcus elongatus* PCC 7942, it was also shown that the ability of SbtB to form a complex with SbtA depends on cAMP and other adenylnucleotides. While higher levels of either ATP or cAMP disturb the complex formation, both, AMP and ADP support the SbtA-SbtB complex formation (Förster et al. 2021) as has been shown in *Synechocystis* (Selim et al. 2018). Despite these detailed insights, the physiological function of a presumably inhibitory interaction of SbtB on the activity of SbtA remains puzzling. The SbtA-SbtB interaction occurs under low Ci conditions, when maximum activity of SbtA is required, whereas the phenotype of *sbtB*-deficient mutants suggest a positive role of SbtB on Ci uptake. Altogether, SbtB appears to play a more sophisticated role than just inhibiting bicarbonate uptake by SbtA, resembling the versatility of canonical PII signaling proteins (Forchhammer et al. 2022). Moreover, a role of SbtB to switch off CCM activity in the dark via phytochrome-mediated signaling has been proposed (Oren et al. 2021).

### SbtB possesses redox-regulated apyrase activity

A striking feature of SbtBs from *Synechocystis* and *Anabaena* sp. PCC 7120 is the aforementioned C-terminal extension that contains a highly conserved C_105_GPxGC_110_ motif, which is widespread among SbtB proteins (Selim et al. 2018). This extension forms a small hairpin loop, in which a Cys-disulfide bridge is formed between the Cys_105_ and Cys_110_ (Fig. 3B). It turned out that this motif is involved in the atypical ATP/ADP diphosphohydrolase (apyrase) activity of SbtB, converting ATP to ADP and further to AMP. This apyrase activity is modulated by the redox-state of this C-terminal motif, which therefore, was termed “R-loop” standing for **R**edox-regulated loop (Selim et al. 2023a). Structural analysis clarified the mechanism by which the redox-state of the R-loop affects the SbtB apyrase activity. The basal part of the T-loop, which is involved in binding of ATP and ADP, sterically communicates with the R-loop. In the ATP-protecting conformation, the basal part of the T-loop can wrap around and coordinate the β- and γ-phosphates of ADP/ADP via R_43_xxR_46_ motif (Fig. 3C). This structure was seen when the R-loop was in the reduced state and disordered. However, when the R-loop gets oxidized and forms the disulfide bridge, it adopts a folding that is in conflict with the ATP-protecting structure of the T-loop. As a consequence, the γ-and β-phosphates of ATP and ADP are forced into a highly strained conformation, exposing the phosphates to hydrolytic attack. Thus, the incompatibility of the R-loop folding with the T-loop folding seems to promote the ATP/ADP hydrolysis, while the reduced and thus unfolded R-loop allows a tighter binding and thus stabilization of ATP/ADP binding. A mutation analysis of either T-loop arginines (Arg_43_ or Arg_46_) or R-loop cysteines (Cys_105_ or Cys_110_) into alanine abolished the SbtB apyrase activity, supporting the importance of both R-loop and T-loop for the nucleotides hydrolysis (Selim et al. 2023a).

The SbtB redox-switch appears to be regulated by thioredoxin TrxA, indicating that it is connected to cellular metabolism (Selim et al. 2023a). *Synechocystis* mutants containing SbtB variants with altered R-loops were constructed, in which the R-loop was either completely deleted or kept permanently in a reduced-mimic state. Physiological analysis of those mutants indicated that a functional R-loop is needed for full activation of Calvin–Benson cycle, proper Ci acclimation, and diurnal growth (Selim et al. 2023a). These findings support the notion that the SbtB R-loop has a broader impact on the *Synechocystis* physiology and suggests that the SbtB redox-switch may serve to coordinate SbtA-SbtB complex formation or other yet unidentified targets in response to the diurnal day-night cycle. Collectively, it appears that SbtB is a sensory module dynamically switching between different adenyl-nucleotide-binding states via slow apyrase activity, which is regulated by the R-loop in response to the cellular redox-state depending on the photosynthetic electron transport chain. Notably, several of enzymes of the Calvin–Benson cycle in cyanobacteria and plants evolved analogous redox-regulated C-terminal extensions formed by C(V/I)VxVC motifs, as the SbtB R-loop, implying an evolutionary conserved role for such redox-motifs in regulation central carbon metabolism in photosynthetic organisms (e.g. Gurrieri et al. 2021).

### c-di-AMP is needed for diurnal growth of cyanobacteria

To reveal the role of SbtB as a possible c-di-AMP receptor, an unbiased search for c-di-AMP binding proteins was performed by affinity chromatography in *Synechocystis* (Selim et al. 2021b). Among a dozen enriched proteins, SbtB was the most abundant one. Furthermore and similar than known form other bacteria, several transporters involved in potassium (KtrA, TrkA, and MthK), sodium (NhaS5 and NhaS2), and magnesium (MgtE) ion homeostasis were identified as putative c-di-AMP receptors. The possible role for binding of c-di-AMP to the Na^+^-gradient restoring Na^+^/H^+^ antiporters, particularly NhaS5, is likely regulating the Na^+^-homeostasis and maintaining the Na^+^-motif force at the cytoplasmic membrane that is required for the Na^+^-dependent bicarbonate transporters SbtA and BicA (Fig. 2). In addition, Mg^2+^-homeostasis is of particular importance for the photosynthetic lifestyle of cyanobacteria, as it is the central ion in the chlorophylls and is required for the maintenance thylakoid membrane integrity (Pohland and Schneider, 2019). By perturbing the cellular levels of c-di-AMP through overexpression of either diadenylate cyclase (*sll0505, dacA* gene) or PDE (*slr0104*) in *Synechocystis*, Agostoni et al. (2018) also revealed its role in salt acclimation and osmotic stress response. Altogether, these results implied that c-di-AMP signaling in cyanobacteria partially matches the canonical functions known from Firmicutes in regulating ion homeostasis and osmotic stress response (Stülke and Krüger 2020; Zarrella and Bai 2021).

But, c-di-AMP plays additional roles that appear to be unique to cyanobacteria. Initially, Rubin et al. (2018) found that c-di-AMP is required for nighttime survival in *Synechococcus elongatus*, a novel role for c-di-AMP in prokaryotes. The inability to survive nocturnal dormancy was attributed to increased oxidative stress during the nighttime periods in the absence of the second messenger. The identification of SbtB as a prominent c-di-AMP receptor unique for cyanobacteria suggested that these two factors might be connected with nighttime survival. Indeed, the *Synechocystis* SbtB deficient mutant showed a similar inability in diurnal growth as a *dacA* mutant (Selim et al. 2021b). To further elucidate the mechanistic link between c-di-AMP and SbtB for nighttime survival, co-immunoprecipitation and pull-down experiments were performed with SbtB in the absence or presence of c-di-AMP. Thereby, several enzymes of the glycogen metabolic machinery were detected. Among them, the glycogen branching enzyme GlgB showed the strongest enrichment. The interaction between GlgB and SbtB was further confirmed by bacterial two hybrid analysis and by biophysical method using purified proteins. In agreement, *sbtB* as well as *dacA* mutants showed strongly reduced levels of glycogen, the key storage molecule that ensures energy supply during the dark and that cyanobacteria need to survive dark periods (Gründel et al. 2012). Measurement of c-di-AMP levels during diurnal growth showed a boost in c-di-AMP levels after light switch on, followed by a slow decline, until a minimum is reached at the end of the night. These data imply a model according to which SbtB perceives via c-di-AMP-binding a signal for the start of the day. This leads to activation of glycogen synthesis during the light period via SbtB interaction with the enzymes of glycogen metabolism, in particular GlgB (Selim et al. 2021b). The molecular mechanism, how SbtB modulates glycogen biosynthesis through GlgB interaction, awaits further elucidation. The second messenger c-di-AMP plays also an important role in nitrogen acclimation of *Synechocystis*, because the c-di-AMP free mutant (Δ*dacA*) was not able to recovery from nitrogen-starvation–induced chlorosis, which is consistent with upregulation of the *dacA* gene under resuscitation conditions (Klotz et al. 2016; Selim et al. 2021b).

Altogether, these studies highlight SbtB as a central switch-point in cyanobacterial cell physiology, integrating not only signals from the energy state (adenyl-nucleotide-binding) and the carbon supply (cAMP binding), but also from the light/dark status reported by the R-loop redox switch and the phase of the diurnal cycle via c-di-AMP binding.

### Second messengers influence the expression of the CCM

The influence of second messengers extends to every part of the cell metabolism, including regulation of gene expression, through transcription factors and other mechanisms. The same appears to be the case in the CCM, where the second messengers cAMP and c-di-AMP play a role in its regulation, possibly through SbtB binding. This has been determined in *Synechocystis* by studying the transcriptome during shifts from high to low CO_2_ conditions using the knock-out mutants *sbtB, cya1* (*slr1991*) for the main adenylate cyclase Cya1, and *dacA* (*sll0505*) for the only di-adenylate cyclase DacA.

Under changing Ci conditions, SbtB proved to play an important role in the proper regulation of gene expression. A large number of genes whose expression has been previously associated with fluctuating Ci levels became deregulated when SbtB was missing (Mantovani et al. 2022). Among the affected genes are multiple components of the CCM, such as the bicarbonate transporters SbtA, the NADH-dehydrogenase 3 and the transcription factor NdhR, one of the main regulators of Ci acclimation, which are all significantly downregulated after the shift to low CO_2_ conditions. The upregulation of several CCM-related genes in the *sbtB* mutant under high CO_2_ conditions, possibly linked to the lowered expression level of the repressor NdhR, correlates well with the observation that the absence of the regulatory protein SbtB causes the cells to find themselves in a low CO_2_ pre-acclimated state even under high CO_2_ conditions (Selim et al. 2018; Mantovani et al. 2022).

The second messenger cAMP proved itself to play a role as a high CO_2_ signal. In fact, the number of de-regulated genes in the absence of the main adenylate cyclase is much higher under high than low CO_2_ conditions. However, while cAMP does seem to function as a high CO_2_ signal, its role in the regulation of the CCM expression appears to be less relevant, since not many Ci-related genes were affected (Mantovani et al. 2022).

The second messenger c-di-AMP, however, appears to be more important than cAMP in the regulation of CCM genes. When the di-adenylate cyclase of *Synechocystis* is knocked-out, many components of the CCM such as the bicarbonate transporters SbtA, BCT1, and BicA or the NADH dehydrogenases 2 and 3 are upregulated even under high CO_2_ conditions, whereas in wild-type cells they are only induced under low CO_2_. Interestingly, the Na^+^-gradient restoring antiporter NhaS6 (*sll0556*), which is potentially needed for the function of the Na^+^-dependent bicarbonate transporters (Fig. 2), is also upregulated in *dacA* mutant. The *nhaS6* expression has been previously shown to be transcriptionally regulated in response to the salt stress in *Synechocystis* (Klähn et al. 2021). Thus, the functional link between c-di-AMP and NhaS6 to either osmotic stress or CCM, awaits further investigation. While not all the regulatory components have been identified yet, these results already give a strong indication of the importance of second messengers in the transcription regulation of the CCM (Mantovani et al. 2022). These conclusions were further reinforced by studying the CCM activity via measuring the Ci-uptake affinity in the *dacA* and *cya1* mutants acclimated to either high or low CO_2_ conditions. These experiments showed that the absence of DacA impacted CCM activity while Cya1 deletion did not (Mantovani et al. 2022).

## Conclusions

Even though only 10 years have passed since SbtB has been discovered as a PII-like protein, it has been clearly shown that SbtB plays an important role in the regulation of the primary carbon metabolism and CCM among cyanobacteria. Meanwhile, various SbtB interaction partners have been identified and the modification of interaction by effector molecules, including second messengers, has been determined. According to these studies, SbtB surpasses the versatility of canonical PII signaling proteins in terms of signal perception highlighting the amazing regulatory potential of small regulatory proteins of the PII superfamily (Forchhammer et al. 2022). However, further studies are required to mechanistically understand the regulatory network of SbtB interaction with yet unidentified targets as well as the interplay between the various adenyl-nucleotides on SbtB sensing.

**Figure 4.**
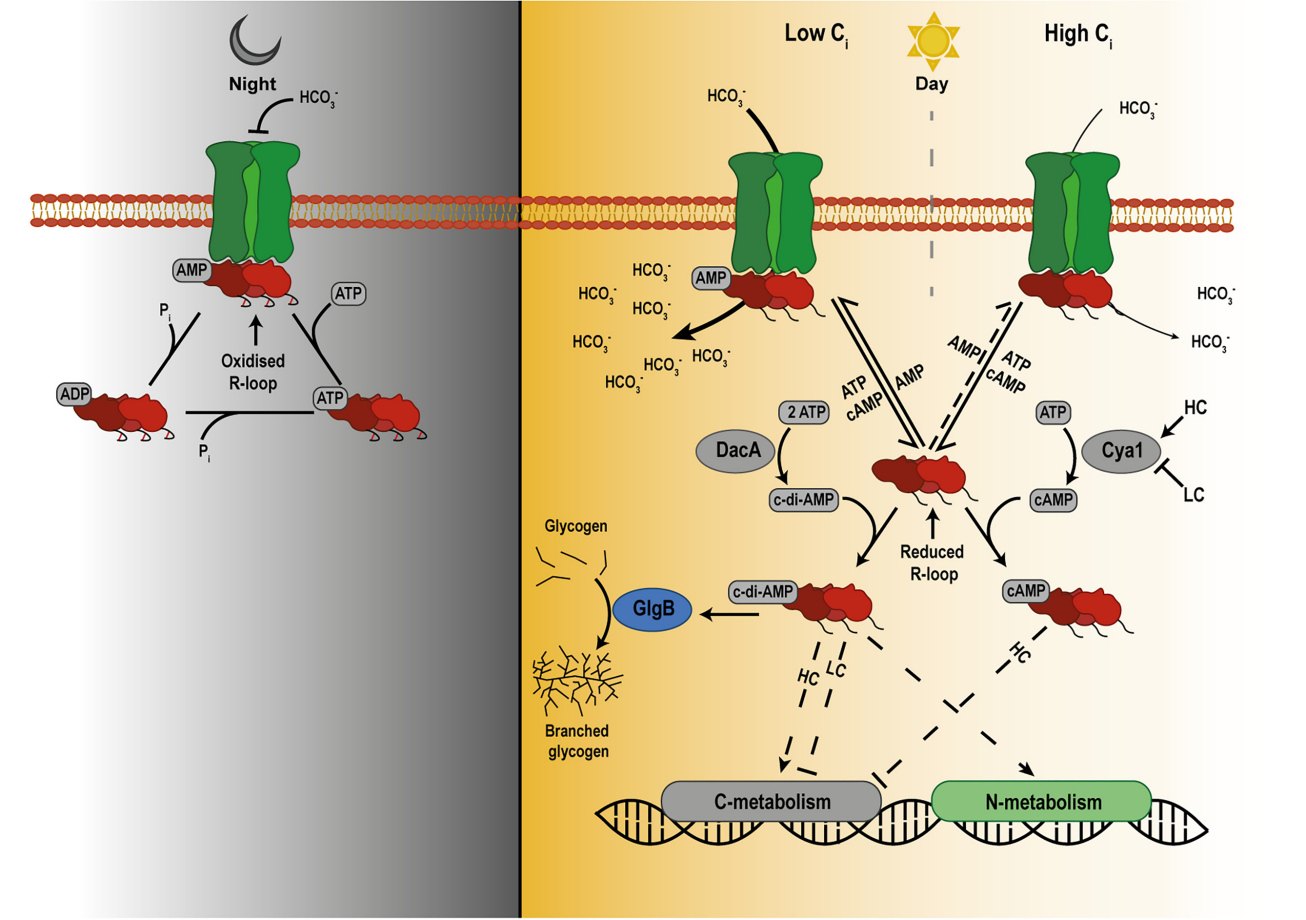
Schematic representation of the regulatory mechanism of SbtB and second messengers in S*ynechocystis* sp. PCC 6803. On the left, the mechanism during the night is shown. The absence of photosynthesis causes the cell to be in an oxidized state, leading the R-loop of SbtB (shown in red) to be oxidized (note the closed drawing of the SbtB tail), activating its apyrase (ATPase/ADPase) activity to reach to the AMP-state, which stabilizes SbtA-SbtB complex formation. SbtB’s role is probably to prevent wasteful bicarbonate uptake in the night by interacting with SbtA (shown in green). The night-active ATPase/ADPase activity keeps SbtB constantly in the AMP state, maintaining its inhibitory function. On the right, the mechanism during the day is shown in cells exposed either to low or high Ci conditions. Under photosynthetic conditions, the R-loop becomes reduced, inhibiting SbtB apyrase activity. Now, SbtB responds to steady state level changes of the various adenyl-nucleotides. Low CO_2_ conditions correlate with increased AMP levels and low cAMP levels, conditions that favor SbtA/SbtB complex formation. Despite being complexed by SbtB, the bicarbonate transporter SbtA is active and highly expressed and thus, the intracellular concentrations of inorganic carbon (Ci) increase. When exposed to high CO_2_ conditions, the bicarbonate transporter SbtA is inactivated probably involving changes in the effector binding state of SbtB mainly via cAMP. Apart from the regulation of SbtA during the day, SbtB performs other regulatory functions. Via the interaction with c-di-AMP, it modulates the activity of the glycogen branching enzyme GlgB, to regulate the synthesis of glycogen. The changing c-di-AMP concentrations also perform a regulatory role on the gene regulation of the CCM, through interaction with SbtB and other unidentified transcription factors. cAMP concentrations increase upon entering high CO_2_ conditions, and causes the inhibition of the CCM through the action of SbtB and other regulators, likely including the transcription factor SyCRP.

Figure 4 summarized our current knowledge of the known regulatory functions of SbtB and of the second messengers capable of SbtB-binding in the CCM. During the day, ATP/AMP- and cAMP-binding to SbtB seem to influence bicarbonate transport in response to fluctuating Ci conditions. The detailed mechanism, how SbtB modulates the activity of SbtA, remains to be elucidated. Moreover, the SbtB-cAMP complex is also affecting gene expression of CCM related genes if the change in carbon availability endures for longer time intervals. The second messenger c-di-AMP, instead, while possibly playing a role in tuning SbtA-SbtB interaction, mostly affects the regulation of the glycogen branching enzyme GlgB, to convert the excess fixed carbon to glycogen. During the night, the apyrase activity of SbtB comes into play, where bicarbonate transport becomes undesired, leading SbtB to increase its binding to SbtA and prevent unneeded bicarbonate from being taken up.

While SbtB’s regulatory function appears to be important in the regulation of the CCM, it is not the only factor controlling the CCM activity. Despite that many interactions in the regulatory network of the CCM have already been identified, the complex interactome is not mechanistically understood. For example, the exact role of SbtB’s interaction with various adenylnucleotides, including cAMP and c-di-AMP in the regulation of the CCM and associated carbon metabolism requires further investigations. Moreover, the specific effects of SbtB interaction with SbtA on the transport activity of the latter remains an open question, i.e. in which conformation the interaction is inhibitory as observed in the heterologous host *E. coli* or enables SbtA-mediated bicarbonate uptake as observed in the illuminated *Synechocystis* cells under low Ci conditions. Other open questions remain with regard to the regulatory role of SbtB on gene expression and further processes distinct from bicarbonate transport via SbtA. For example, in analogy to the canonical PII protein, we expect SbtB to affect the activity of transcription factors, either by direct interaction or indirectly through small mediator proteins, as has been exemplified for NtcA by the mediator protein PipX (Forchhammer et al. 2022). This would allow SbtB in a PII-like manner, by perceiving the various effector molecules, including second messengers, to transduce the Ci and energy status on gene expression. Moreover, the detected interaction with other transporters necessary for salt acclimation needs more detailed investigations. Finally, the proposed regulatory role of SbtB during the night, and the relation between SbtB and light receptors, both need further research to gain a more complete view on the regulatory role of SbtB. These avenues of research promise an exciting future regarding a comprehensive understanding of SbtB and second messengers in cyanobacteria.
